# Neuroendocrine Sarcoidosis: A Rare Bird

**DOI:** 10.7759/cureus.82635

**Published:** 2025-04-20

**Authors:** Somia Hassane, Sadia Tariq, Cecil Eboh

**Affiliations:** 1 Medicine, University Hospital Sussex NHS Foundation Trust, Chichester, GBR; 2 Acute Medicine, University Hospital Sussex NHS Foundation Trust, Chichester, GBR; 3 Internal Medicine and Endocrinology, University Hospital Sussex NHS Foundation Trust, Chichester, GBR

**Keywords:** endocrine manifestations, extrapulmonary sarcoidosis, multiorgan sarcoidosis, neurosarcoidosis, pulmonary sarcoidosis

## Abstract

Sarcoidosis is a systemic auto-inflammatory disease of unknown etiology. It is characterized by granuloma formation and can affect any organ. While it commonly affects the lungs and the lymphatic system, it can also involve the central nervous system, leading to neurosarcoidosis or the endocrine system and subsequent multiple endocrinal pathologies. Neurosarcoidosis can affect any part of the nervous system and therefore manifests with variable symptoms mimicking other neurological pathologies. Any endocrine gland can be infiltrated by sarcoidosis; however, the pituitary gland and the hypothalamus are the most frequently infiltrated, while thyroid sarcoidosis cases remain rare. Only a biopsy of a suspected lesion is considered to be a definitive diagnostic method, which makes diagnosing central nervous system sarcoidosis particularly challenging. This case report highlights the challenges of diagnosing neuroendocrine sarcoidosis as the first presentation.

## Introduction

Sarcoidosis is an inflammatory disease characterized by non-caseating granulomas. Around 1 in every 10,000 people have sarcoidosis in the UK, with 80% of cases being between 37 and 65 years of age. Up to 94% of patients with sarcoidosis have pulmonary involvement [1] and first present with respiratory-related symptoms, making it difficult to suspect in patients presenting with neuroendocrine sarcoidosis as a first presentation.

Neurosarcoidosis can occur in up to 15% of sarcoidosis cases, making it a rare condition with a prevalence rate of 20 per million [2,3]. Although rare, hypothalamic-pituitary involvement is the most common among the endocrine glands to be affected [4]; on the other hand, thyroid sarcoidosis is only reported in 4.2% to 4.6% of sarcoidosis cases [5].

Histological diagnosis remains the gold standard investigation for a definite diagnosis; however, it is avoided in cranial involvement due to the risks associated with such an approach [6]. A highly probable diagnosis is reached when other differentials are ruled out, symptoms are suggestive, and evidence of central nervous system (CNS) inflammation is present on imaging or cerebrospinal fluid. The multisystem nature of sarcoidosis requires the involvement of multiple specialists among other members of the multidisciplinary team (MDT) to manage the disease. This case report presents the case of a patient with neurosarcoidosis who presented to our local hospital.

## Case presentation

A 52-year-old female was brought to the Emergency Department with a one-day history of worsened confusion, fever, vomiting, inability to mobilize, and urinary incontinence; she was generally feeling unwell. The patient had a six-month history of dizziness, loss of balance, headaches, memory issues, blurred vision, and intermittent diplopia. She also had a history of rosacea, back pain, osteoarthritis, and type 2 diabetes and was only on desogestrel (for hormonal replacement therapy) and omeprazole. She was a non-smoker and took alcohol socially. She worked as an administrator and lived with her husband and was fit and independent. There was no history of travel or exposure to the usual dust molds or hay.

On examination, she was conscious but confused, could not recall the sequence of events, was lethargic, and was unable to sit up in bed without support. She was hemodynamically stable with a temperature of 38.3°C. Neurological examination revealed an abbreviated mental test score of 3 out of 10, with no nuchal rigidity, photophobia, or neck stiffness. Her speech was normal, her pupils were equal and reactive, and she had a normal cranial nerve examination. Her tone, power, reflexes, and sensation were all normal in all four limbs. Worthy of note is that she had no dysdiadokokinesia or abnormal finger nose test; however, she had a broad-based gait and imbalance on walking. Finally, her chest, cardiovascular, and abdominal examinations were all unremarkable. Table 1 shows the patient’s blood tests, and Table 2 shows her venous blood gas results.

**Table 1:** Blood results HBA1c, glycated hemoglobin; CRP, C-reactive protein; ACE, angiotensin-converting enzyme; TSH, thyroid-stimulating hormone; T4, thyroid hormone; FSH, follicle-stimulating hormone; LH, luteinizing hormone; IGF, insulin-like growth factor; OCP, desogestrel

Blood work-up	Result	Normal range
Ketones	1.4 mmol/L	0.6 mmol/L
HBA1c	13.4%	<6.5%
CRP	6 mg/L	0–5 mg/L
Neutrophils	15.7 mg/L	2–7 10^9^/L
White cells	17.9 10^9^/L	4–10 10^9^/L
ACE serum	17.5 u/L	20–70 u/L
Blood culture	No growth	
TSH	1.56 mU/L	0.3–4.9 mU/L
T4	14 pmol/L	9–19 pmol/L
FSH	1.5 IU/L (on OCP)	Postmenopausal >25 IU/L
LH	0.1 IU/L (on OCP)	Postmenopausal >25 IU/L
Cortisol	614 nmol/L	150–600 nmol/L
Prolactin	513 mU/L	109–557 mU/L
IGF	16 nmol/L (on OCP)	9.5–27.8 nmol/L

**Table 2:** Venous blood gas results pH, potential of hydrogen; HCO_3_, bicarbonate; BG, blood glucose; BE, base excess

Venous blood gas	Result	Normal range
pH	7.43	7.350–7.45
HCO_3_	23 mmol/L	22–29 mEq/L
BG	29.6 mmol/L	3.9–5.8 mmol/L
Lactate	2.6 mmol/L	05–1.6 mmol/L
BE	4.9 mmol/L	− 2 to + 2 mmol/L

**Figure 1:**
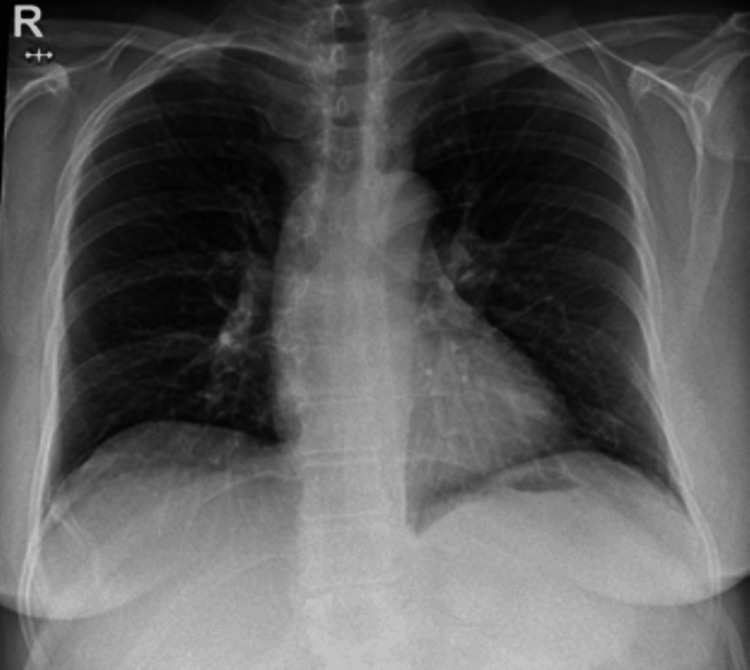
Chest radiograph

Chest X-ray (CXR) was performed, which showed no abnormal finding (Figure 1).

**Figure 2:**
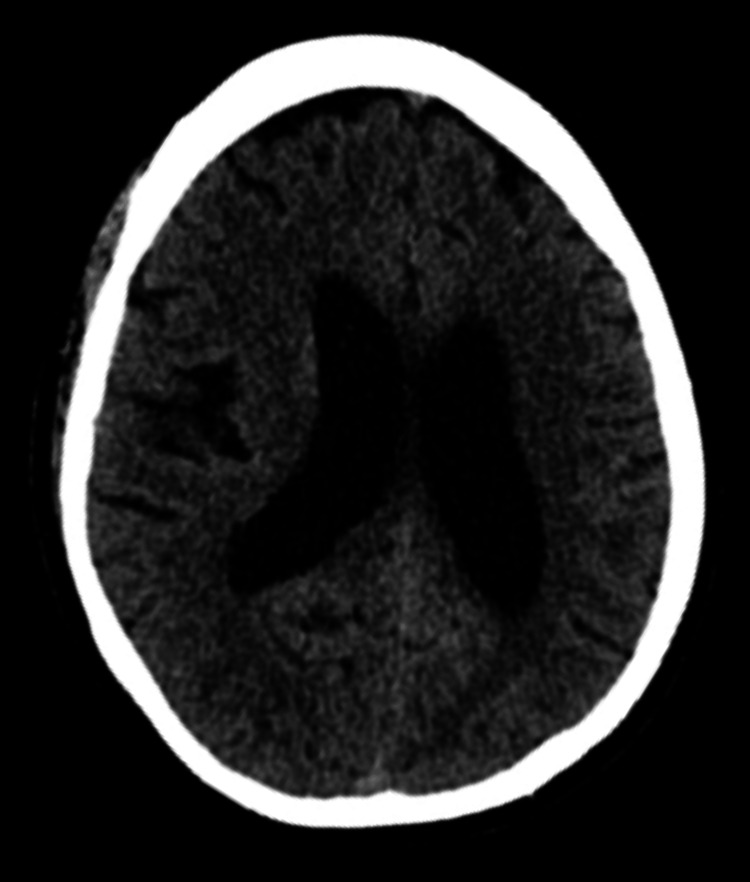
Computed tomography scan of the head

Computed tomography scan (CT) of the head was reported as unremarkable, as shown in Figure 2.

She was treated with intravenous ceftriaxone and acyclovir for a possible meningoencephalitis. The neurologist reviewed the patient and advised for a magnetic resonance imaging (MRI) of the head and a lumbar puncture (LP). The results of the LP are shown in Table 3.

**Table 3:** Cerebrospinal fluid results OP, opening pressure; LDH, lactate dehydrogenase; ACE, angiotensin-converting enzyme; VZV, varicella zoster virus; HSV, herpes simplex virus; OGB, oligoclonal bands

Lumbar puncture	Result	Normal range
OP	20.5 mmH_2_O	10–20 cmH_2_O
Lactate	12.8 mmol/L	1.2–2.1 mmol/L
Glucose	2.2 mmol/L	2–4.5 mmol/L
Protein	Not reported	
LDH	37 IU/L	<40 IU/L
ACE	5.4 uM/min/L	0-1.2 uM/min/L
White cells	67/cubic mm (100% mono)	0–5/cubic mm
Gram stain	No organism	
VZV DNA	Not detected	
Enterovirus RNA	Not detected	
HSV DNA	Not detected	
OGB	Not reported	
Culture	No growth	

**Figure 3:**
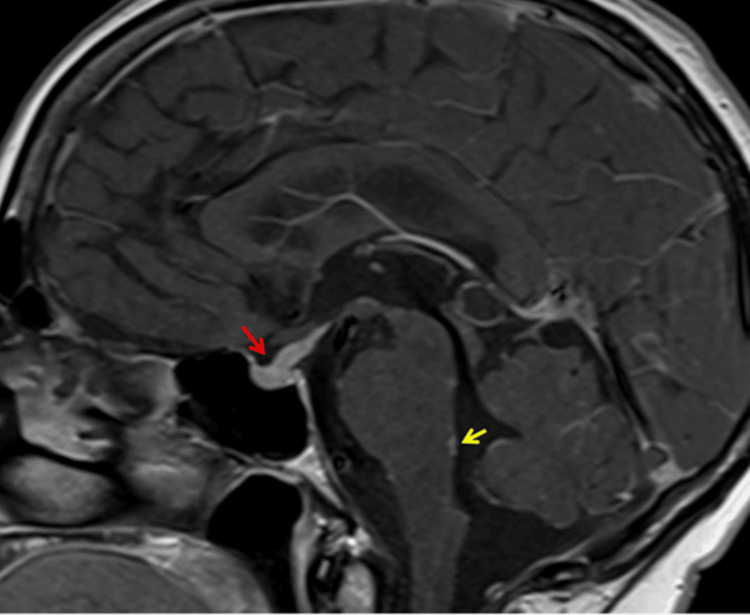
Magnetic resonance imaging of the pituitary Red arrow indicates thickening of the pituitary stalk, and the yellow arrow indicates leptomeningeal post-contrast enhancement.

MRI of the head was concerning for a possibility of normal pressure hydrocephalus and thickening of the pituitary stalk. Therefore, the neurologist advised MRI of the sella turcica with contrast, a hormone profile review, and an endocrine review. MRI of the head and pituitary with contrast showed pituitary hypophysitis and leptomeningeal enhancement. MRI of the pituitary showed abnormal thickening and enhancement of the pituitary stalk, both third cranial nerves, the surfaces of the hypothalamus and third ventricle walls, surface of the dorsal pons, and cerebellar folia, indicating widespread leptomeningeal enhancement (Figure 3).

**Figure 4:**
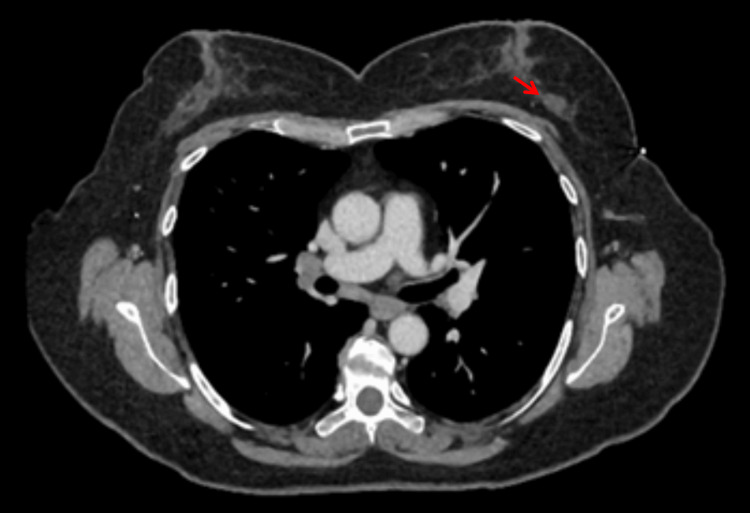
Computed tomography scan of the chest Red arrow indicates a 17-mm left outer breast nodule

**Figure 5:**
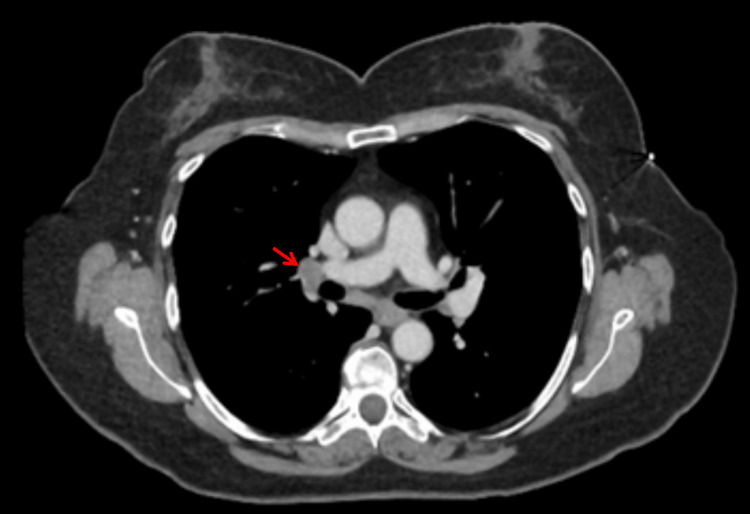
Computed tomography scan of the chest Red arrow indicates a right hilar lymph node

**Figure 6:**
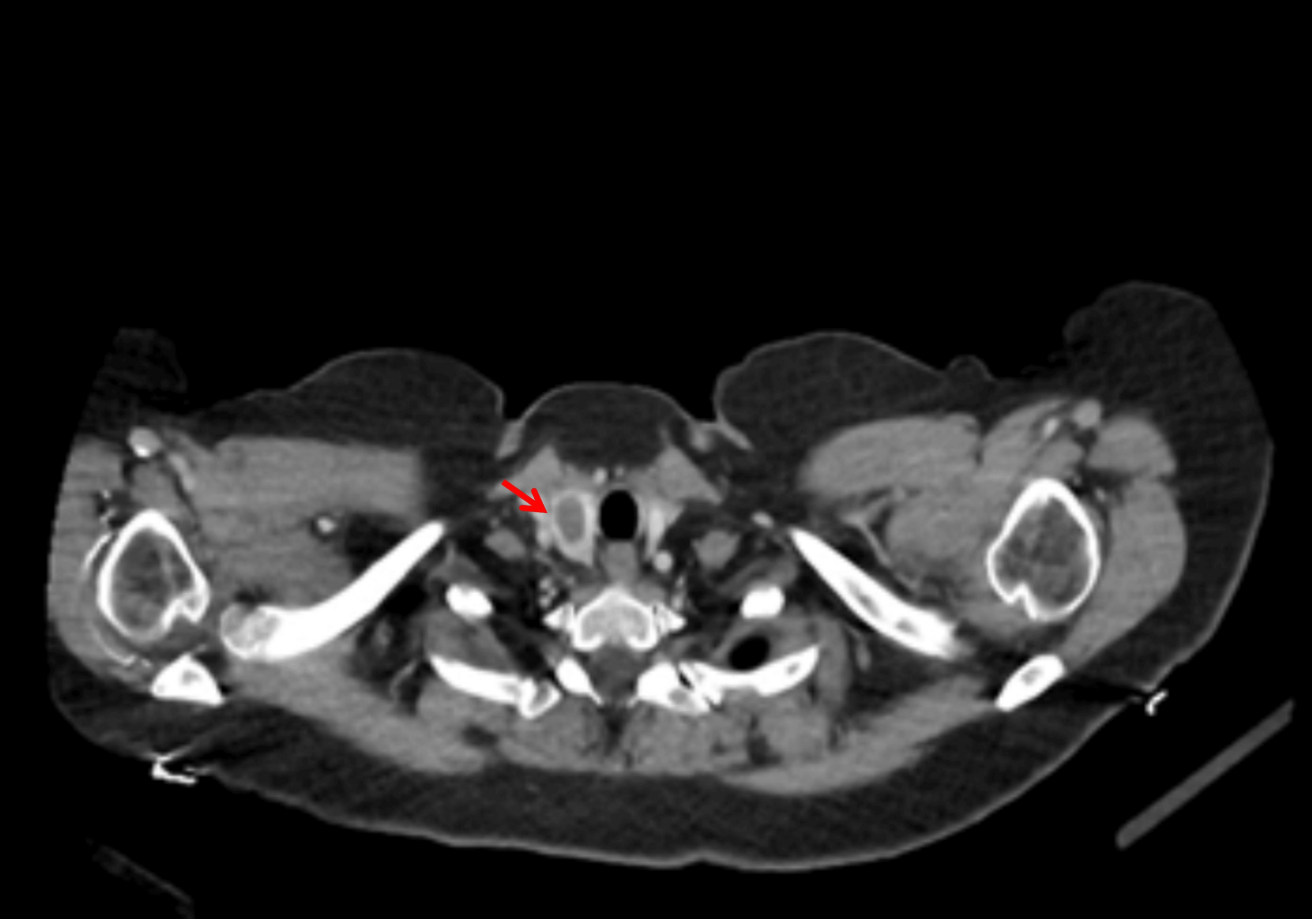
Computed tomography scan of the chest Red arrow indicates an enlarged right thyroid lobe

The case was then discussed at the pituitary multidisciplinary team (MDT) meeting, which advised to perform a CT of the thorax, abdomen, and pelvis to rule out a malignancy. The CT of the thorax, abdomen, and pelvis showed multiple enlarged lymph nodes, specifically a 17-mm left outer breast nodule, 8-mm left axillary lymph node, a right hilar node measuring 13 mm, a left hilar node measuring 9 mm, and a subcarinal node measuring 8 mm. Multiple small-volume mediastinal nodes were also noted but they were not enlarged ad per CT criteria. Also, an enlarged right lobe of the thyroid gland was also visible. Important findings on the CT of the thorax, abdomen, and pelvis are highlighted in Figures 4-6.

**Figure 7:**
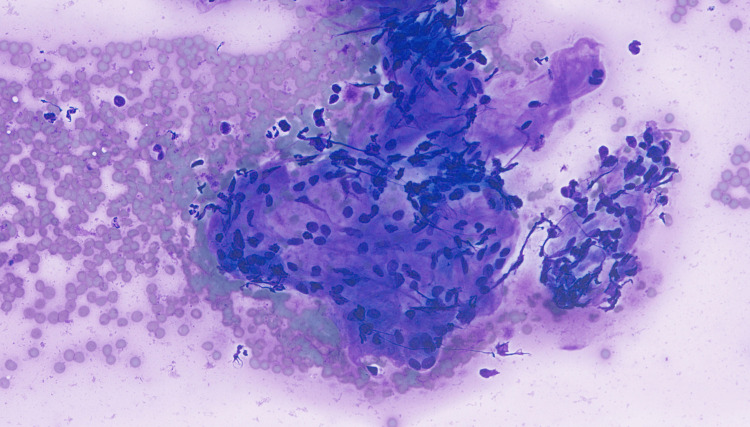
Fine needle aspirate of the right lobe of the thyroid gland Cellular aggregate consistent with a sarcoid granuloma was present in a fine needle aspirate of the right lobe of the thyroid gland (Romanowsky stain, original magnification x400).

Endocrine advised referral to the endocrine and pituitary MDT and an ultrasound of the thyroid, which revealed two well-defined right-sided thyroid nodules. Fine needle aspiration of the thyroid initially reported macro- and micro follicular aggregates of epithelium, which exhibited widespread oncocytic metaplasia suggestive of follicular neoplasm; however, after further discussion, it was amended and reported as showing the presence of abundant thyroid epithelium with aggregates, consistent with sarcoid granulomas (Figure 7).

**Figure 8:**
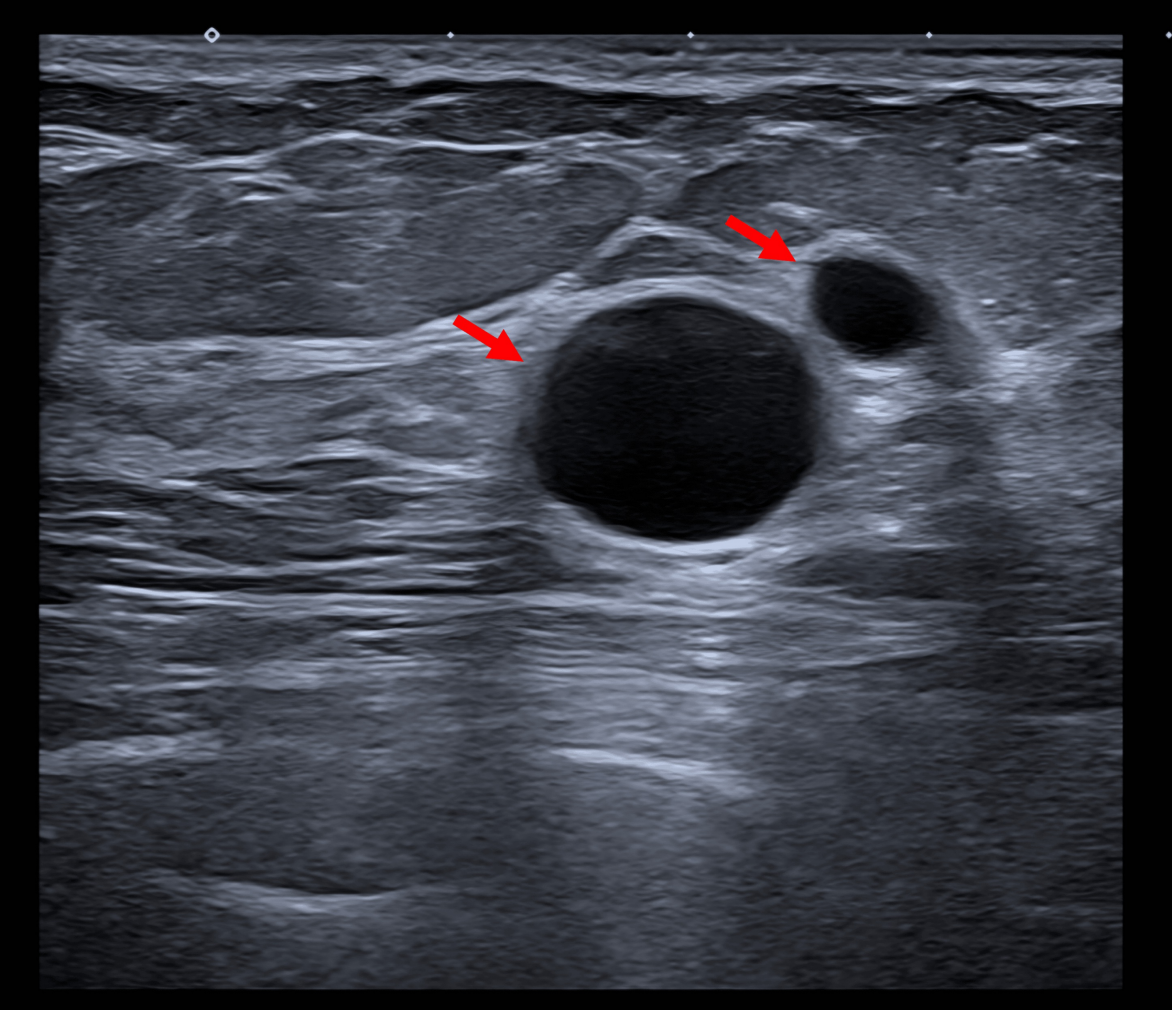
Left breast ultrasound Red arrows indicate multiple benign cysts

In view of the left hilar lymphadenopathy and mediastinal lymph nodes, the case was discussed at the respiratory MDT meeting and the outcome was to refer for a diagnostic endobronchial ultrasound (EBUS) and biopsy to rule out pulmonary sarcoidosis along with pulmonary function test. Results of EBUS showed non-necrotizing granulomatous lymphadenopathy, which is diagnostic for pulmonary sarcoid. The patient was started on prednisolone 80 mg once a day with plan to gradually reduce. The case was also discussed at the breast MDT meeting in view of the left breast nodule, and the triple assessment results were unremarkable. Figure 8 below shows benign cysts seen on left breast ultrasound.

An echocardiogram was performed to rule out cardiac involvement, and findings were also normal (Video 1).

**Video 1:** Echocardiogram

As for her follow-up as an outpatient, she was seen by both neurology and respiratory teams, and the patient reported significant improvement in her symptoms.

## Discussion

Sarcoidosis is a multisystem inflammatory disease of unknown etiology. It manifests as non-caseating granulomas in the affected organs in the absence of defined toxic or infective trigger. Most specialists agree that around 1 in every 10,000 people have sarcoidosis in the UK, with 80% of cases being between 37 and 65 years of age. The average reported age was 50, with the suggestion that it is slightly more prevalent in females [1,3]. Recent cohorts of sarcoidosis patients showed that sarcoidosis was found to be the most frequent cause of death, compared to the general population, with an average life span of six years less than the general population [7].

A number of studies have linked sarcoidosis with exposure to silica, mold, aluminum, and metal dust [8]. There are theories regarding a link between sarcoidosis and cytokines, including Th1, IL-2, IL-6, IL-8, IL-12, IL-18, IL-27, interferon gamma, and tumor necrosis factor [9]. It is also worth mentioning that sarcoidosis has been reported by multiple studies to have a genetic component, with significant associations between sarcoidosis and the HLA class II (DRB1 and DQB1) alleles [10].

Our patient had a triad of neurosarcoidosis (with pituitary involvement), pulmonary sarcoidosis, and thyroid involvement.

Neurosarcoidosis (with pituitary involvement)

The first mention of the disease affecting the nervous system was in 1905 [11]. The presentation varies depending on the affected part of the nervous system, with possible symptoms including aseptic meningitis, conus or quad equine syndrome, cranial neuropathy, focal neuropathy, multifocal, diffuse encephalopathy, hypothalamic and pituitary dysfunction, myelopathy, raised intracranial pressure, seizures, spinal cord radiculitis, uveoparotid fever, and vascular syndromes [12-16].

There is no single diagnostic test for sarcoidosis; rather, diagnostic criteria are used, including the exclusion of other differential diagnoses, clinical and radiological features, and histological findings on a biopsy. The diagnosis of neurosarcoidosis is classified into the following [17]: definite (a positive biopsy, plausible symptoms, and the absence of any other explanation for the symptoms), probable (evidence of inflammation of the CNS [MRI and CSF], plausible symptoms, and the absence of any other explanation for the symptoms), and possible (plausible symptoms, the absence of any other explanation for the symptoms, and the other criteria not present).

Investigation in neurosarcoidosis usually starts with a CXR [18] since intrathoracic disease is more common, followed by a high-resolution CT thorax as it is more sensitive and may show nodules or hilar adenopathy. This can be followed by bronchoalveolar lavage, which usually shows lymphocytosis with a high CD4:CD8 ratio. Bronchoalveolar lavage can also aid in determining a biopsy site.

As for CNS imaging, it starts with a CT, which may show enhancing nodules, granulomatous lesions, hydrocephalous, or calcifications. Although nonspecific, MRI is more sensitive than CT and can detect parenchymal lesions, meningeal involvement, hypothalamic involvement, optic nerve enlargement, nonspecific leptomeningeal and parenchymal enhancement, granulomatous vasculitis and nonspecific white matter, and leptomeningeal or parenchymal changes [19].

Once the CSF is obtained, it typically shows inflammatory changes such as elevated protein, pleocytosis, low glucose, and, to a lesser extent, oligoclonal bands. However, in some cases, especially isolated nerve palsies, the CSF can be normal [14,15.

Naturally, tissue diagnosis depends on the affected systems. It can be via lymph node biopsy, endobronchial biopsy, or a skin lesion biopsy. For isolated neurosarcoidosis, tissue can be acquired via a nerve biopsy, a muscle biopsy, or even a brain biopsy [15].

Typical histology findings are multiple non-caseating granulomas [20,21], which appear as a well-organized area of granulomatous inflammation without any necrosis, formed by epithelioid cell aggregates, lymphocytes, leukocytes, giant cells, mast cells, and plasma cells.

Pituitary Sarcoidosis

When it comes to endocrine involvement, the pituitary gland and the hypothalamus are the most frequently infiltrated endocrine glands by sarcoidosis [4] it could occur in a previously known case, or it could precede the diagnosis.

As a result of this, the presentation can be variable, ranging from being asymptomatic to causing some of the most serious and dramatic complications of the disease. Diabetes insipidus, hyperprolactinemia, and hypogonadism are the most common endocrine manifestations [22].

To investigate pituitary involvement in sarcoidosis, an MRI of the pituitary is obtained. The radiological findings on MRI can reveal pituitary stalk thickness, involvement of the pituitary gland, and other parenchymal brain or spinal cord lesions mentioned above [22], while histology frequently reveals vascular and perivascular granulomas [4].

Pulmonary sarcoidosis

Pulmonary involvement has a high incidence of 94% in patients with sarcoidosis [1]. Notably, the presentation can be variable and often with a normal chest examination. The most common systemic symptoms are fevers, night sweats, fatigue, weight loss, diffuse myalgias, sarcoid uveitis, or parotitis. The triad of bilateral hilar lymphadenopathy, erythema nodosum, and bilateral ankle arthritis is known as Lofgren’s syndrome. It is important to keep in mind that patients with node-limited disease can be asymptomatic; however, if there is parenchymal involvement, patients usually develop chest symptoms such as dyspnea, cough, and pleuritic discomfort [23].

When it comes to investigating pulmonary sarcoidosis, all patients with suspected sarcoidosis should undergo a CXR. If they have typical findings on a radiograph with a typical clinical presentation (e.g., in the context of Lofgren’s disease), then a CT scan may not be necessary as long as patients are followed up in clinic with a repeat CXR within three months and a CT scan is performed if circumstances change. Multidisciplinary review of chest imaging is recommended to determine the need for a confirmatory bronchoscopy or biopsy [18,23].

Sarcoidosis can be staged from 0 to IV based on radiological findings where stage 0 shows a normal chest radiograph, stage I shows enlarged nodes only, stage II shows enlarged nodes and parenchymal changes, stage III shows parenchymal changes without enlarged nodes or fibrosis, and, finally, stage IV shows fibrosis [23].

As for bronchoscopy, patients with mainly lymph node disease should undergo an endobronchial ultrasound EBUS. Patients with mainly parenchymal disease should have transbronchial biopsies as per recommendations from the British Thoracic Society, which has released an algorithm on their public website for guidance on further investigations of pulmonary sarcoidosis [23].

Thyroid sarcoidosis

Thyroid sarcoidosis has a rare incidence, reportedly seen in 4.2% to 4.6% of sarcoidosis cases (postmortem) [5]. When it comes to presentation, almost all cases are euthyroid. However, sarcoid infiltrates can cause diffuse goiter and rarely a solitary thyroid nodule [24]. These infiltrates can result in symptoms of thyroid hypofunction, which is the most common functional abnormality in thyroid sarcoidosis, although rare cases of Graves' disease have also been reported [25].

To investigate thyroid sarcoidosis, thyroid ultrasound should be performed. The results are variable and could show goiter, thyroid nodules, and even a large mass. This is followed by fine needle aspiration and cytology, which shows similar findings to the typical histology findings mentioned previously: polymorphic lymphocytes, few lymphohistiocytic aggregates, scant Hurthle cells, and multinucleated giant cells.

Finally, treatment of sarcoidosis is usually done using a multidisciplinary approach due to the systemic nature of the disease, and multiple specialties usually will coordinate to give the best care.

Treatment of sarcoidosis starts with corticosteroids as the first line. Oral prednisolone is usually started at a higher dose and then gradually reduced and maintained with or without a second agent. In steroid refractory sarcoidosis, second-line treatment includes steroid-sparing immunosuppressive drugs as: azathioprine, cyclosporine, cyclophosphamide, chlorambucil, and methotrexate. Success rates of each treatment are variable, and this is evident in multiple studies [2,26]. Due to the inflammatory nature of the disease and the cytokine activity associated with it, biological agents have also been trailed as third-line agents. Success rates have been variable, with some studies showing no benefit for etanercept [27], while significant benefit was reported in other studies with infliximab [28]. A few studies have also assessed the benefits of using cerebral irradiation therapy [13].

Patients with pituitary disease show radiological improvement to the above treatment options; however, patients rarely recover from hormonal deficiencies and will need replacement accordingly. Hormonal dysfunctions and radiologic outcomes were not correlated [22].

Patients with thyroid sarcoidosis will have periodic clinical and biochemical assessments if they are euthyroid and without complications. Hypothyroid patients will get replacement therapy, thyrotoxic patients will receive anti-thyroid treatment and may require thyroidectomy [24,25].

Prognosis

The five-year follow-up mortality rate is close to 7%. Up to 40% of patients with sarcoidosis develop progressive pulmonary disease, and >60% of sarcoidosis-related deaths are due to advanced cardiopulmonary disease [29].

## Conclusions

This case report highlights the importance of considering this rare pathology among the differential in new neurological cases, especially in young and otherwise fit patients. It is a difficult diagnosis to reach given the wide range of possible presentations as mentioned above. Therefore, it is important to get specialist advice early on to ensure that the appropriate investigations are done to facilitate early treatment. The role of MDT in this case was significant in reaching the correct diagnosis, and it was a collective effort in managing this multisystem disease. There are several options for treatment as described above; thus, follow-up is of valuable importance.
